# Conditions to Consider When Choosing Fillers

**DOI:** 10.1111/jocd.70075

**Published:** 2025-03-10

**Authors:** Gi‐Woong Hong, Jovian Wan, Song‐Eun Yoon, Sky Wong, Kyu‐Ho Yi

**Affiliations:** ^1^ Samskin Plastic Surgery Clinic Seoul Korea; ^2^ Medical Research Inc Wonju Korea; ^3^ Brandnew Aesthetic Surgery Clinic Seoul Korea; ^4^ Leciel Medical Centre Hong Kong; ^5^ Division in Anatomy and Developmental Biology, Department of Oral Biology, Human Identification Research Institute, BK21 Four Project Yonsei University College of Dentistry Seoul Korea; ^6^ You & I Clinic (Mokdong) Seoul Korea

**Keywords:** hyaluronic acid fillers, non‐hyaluronic acid fillers, patient safety, rheology, viscoelasticity

## Abstract

**Background:**

The selection of dermal fillers in aesthetic medicine often relies on factors such as cost, immediate outcomes, and practitioner experience. However, incorporating knowledge of fillers' rheological properties, such as viscoelasticity and cohesiveness, allows for more precise product selection tailored to patient needs and treatment goals while reducing the risk of complications.

**Aims:**

This review aims to summarize essential considerations for filler selection, focusing on rheological properties, safety profiles, and clinical applications. Additionally, it seeks to highlight differences between hyaluronic acid (HA) fillers and non‐HA fillers to guide practitioners in aesthetic procedures.

**Patients/Methods:**

A systematic review was conducted following PRISMA guidelines. Searches across PubMed, Scopus, Web of Science, and the Cochrane Library yielded 619 articles. After duplicate removal and rigorous screening, 50 peer‐reviewed studies were included. Data extraction focused on filler types, rheological properties (e.g., *G*′ and *G*″ values), safety, and efficacy.

**Results:**

HA fillers, particularly monophasic types, exhibit smoother consistency and better cohesiveness, making them ideal for high‐mobility areas like the mouth. Biphasic fillers, with higher viscoelasticity, provide superior lifting capacity for deeper tissue support. Non‐HA fillers, such as poly‐L‐lactic acid and calcium hydroxylapatite, offer longer‐lasting results but require precise techniques due to irreversibility. Proper selection based on filler rheology, target area, and patient needs can mitigate risks such as overfilled syndrome, Tyndall effect, and delayed inflammatory responses.

**Conclusions:**

Understanding the rheological and safety profiles of fillers is essential for achieving optimal aesthetic outcomes. HA fillers are recommended for novice practitioners due to their reversibility, while experienced clinicians may explore non‐HA options. Tailored filler selection based on rheological properties and clinical context ensures safer and more effective treatments.

## 1 Introduction

In the absence of rheological knowledge or information, clinicians frequently base their selection of HA fillers on factors such as product cost, immediate post‐procedural outcomes, patient satisfaction, or the experiences and recommendations of colleagues. However, by integrating rheological knowledge, clinicians can make more informed decisions, thereby minimizing reliance on trial and error influenced by marketing or anecdotal evidence. This informed approach facilitates the selection of fillers that meet essential clinical requirements. Subsequently, considering the patient's facial condition and aesthetic preferences is likely to yield optimal results. This review summarizes the key considerations, including the physical properties of fillers, which are essential for their selection in clinical practice.

## 2 Methods

A systematic review was conducted following PRISMA guidelines to identify studies relevant to the topic, “Conditions to Consider When Choosing Fillers.” Searches were performed across four major databases—PubMed, Scopus, Web of Science, and the Cochrane Library—using a comprehensive search strategy. The search utilized a combination of keywords and Boolean operators, including “hyaluronic acid fillers,” “non‐hyaluronic acid fillers,” “rheological properties of fillers,” “viscoelasticity in fillers,” and “filler safety and efficacy.” Search strings were structured as “(Hyaluronic acid OR HA fillers) AND (viscoelasticity OR rheology) AND (adverse effects OR complications).” The review was restricted to full‐text articles published in English up to January 2025.

The initial search identified 619 articles. After duplicate removal, 421 articles remained for screening. Title and abstract screening excluded 300 articles that were irrelevant to the topic or failed to meet the inclusion criteria. Subsequently, full‐text assessments were performed on 123 articles, resulting in further exclusions due to incomplete data, irrelevance, lack of clinical applicability, or non‐English language. Ultimately, 50 studies met the criteria and were included in the final review.

Eligible studies were required to be published in peer‐reviewed journals, focus on hyaluronic acid (HA) fillers or non‐hyaluronic acid (non‐HA) fillers (e.g., poly‐L‐lactic acid [PLLA], calcium hydroxylapatite [CaHA], polymethyl methacrylate [PMMA]), and address filler properties such as rheology, elasticity, viscosity, or cohesiveness. Studies reporting safety profiles, adverse effects, or efficacy outcomes involving human participants or clinically relevant in vitro analyses were also included.

Exclusions were applied to non‐English publications, studies limited to animal models without clinical relevance, case reports, editorials, and commentaries with insufficient data. Articles discussing fillers unrelated to aesthetic or dermatologic applications, lacking full‐text access, or identified as duplicates across databases were also excluded.

The study selection process was carried out in two stages. First, titles and abstracts were screened for relevance, followed by a full‐text assessment against inclusion and exclusion criteria. Two independent reviewers conducted all evaluations, with disagreements resolved through discussion or consultation with a third reviewer.

Data extraction followed a standardized process, collecting details on authorship, publication year, study location, design, filler types (HA or non‐HA), rheological properties (e.g., *G*′ and *G*" values, cohesiveness), safety profiles, adverse events, and clinical outcomes such as longevity and patient satisfaction. Extracted data were synthesized into key themes, including filler types, rheological characteristics, safety considerations, and adverse effects. This rigorous and methodical approach ensured a reliable and comprehensive analysis of evidence, culminating in the inclusion of 50 high‐quality studies for detailed review.

### 2.1 Hyaluronic Acid Fillers Versus Non‐Hyaluronic Acid Fillers

For novice filler practitioners, it is advisable to initially employ HA fillers that can be dissolved and removed if necessary. Apart from severe side effects due to intravascular injection, HA fillers can be promptly degraded using the enzyme hyaluronidase in the event of complications or unsatisfactory outcomes [1, 2, 3, 4, 5].

Once proficiency in performing safe and effective filler procedures has been achieved, practitioners may consider using longer‐lasting fillers at their discretion. However, fillers composed of substances other than hyaluronic acid cannot be dissolved and removed, necessitating their sparing and cautious use [6, 7].

Irrespective of the type of filler chosen, it is imperative to prioritize safety‐proven products. As previously mentioned, newly developed fillers should be used restrictively and observed in clinical settings for a minimum of 6 months before broader adoption. Managing complications that arise post‐procedure is often more challenging than the procedures themselves, particularly for novice practitioners who may lack experience in handling adverse effects. Therefore, it is prudent to minimize complications stemming from product issues rather than practitioner errors. The most reliable method for ensuring safety is to select fillers that have been extensively used over a prolonged period without major issues and have consistently demonstrated reliable results [8].

### 2.2 Basic Approval Conditions for Hyaluronic Acid Filler Products

When selecting the most commonly used HA fillers, it is imperative to understand the clinical requirements and the basic approval criteria set by regulatory agencies such as the Korean Ministry of Food and Drug Safety (MFDS). Clinically desirable HA fillers must meet specific conditions to ensure both safety and efficacy.

First, HA fillers must be biocompatible and biodegradable, containing no harmful chemicals. They should not provoke severe inflammatory or immunological reactions and must integrate well with human tissue, maintaining stability at the injection site without migration. Most importantly, HA fillers should effectively smooth wrinkles and enhance volume in accordance with the treatment goals. Over the long term, they should preserve their results without significant deformation or degradation [9, 10].

While some of these desirable conditions can only be confirmed through actual clinical use, all products must pass certain laboratory tests prior to market release. Filler manufacturers are required to submit measurements of various parameters, including pH, osmotic pressure, sterility, toxicity, endotoxin levels, heavy metal content, residual cross‐linking agents, and the injection pressure required for the product.

Using a recently released domestic HA filler product as an example, Table 1 outlines the laboratory test items necessary for MFDS approval, the standard values for each item, and the results submitted by the manufacturing company. This information serves as a guideline for understanding the approval process and ensuring the selection of safe and effective HA fillers.

**TABLE 1:** Laboratory test standards and results for HA filler product.

Category	Standard	Result
Appearance	Transparent and colorless viscous liquid with no foreign substances	Satisfied
pH	6.8–7.5	6.83
Residual BDDE	Maximum 2.0 PPM	0 PPM
Heavy metal	Maximum 10.0 PPM	Below 10 PPM
Injection force	Maximum 30 N	14.9 N
Osmotic pressure	200–400 mmol/kg	318 mmol/kg
Volume	Minimum 1.0 mL	1.07–1.09 mL
Septic/toxic	Aseptic/non‐toxic	Satisfied

### 2.3 Quality and Quantity of Raw Materials

When selecting a filler, it is crucial to understand the quality and quantity of the raw materials used in its production. Even if products are manufactured using the same process, differences in raw material quality can significantly affect the frequency of post‐procedure complications. Therefore, it is important to verify the source of the raw materials for each filler product [11, 12].

Globally, there are hundreds of HA filler products, but the molecular weight and concentration of HA used as raw materials do not vary significantly between products. The HA used in HA fillers is typically extracted from bacteria, with a molecular weight generally ranging from 1.5 million to 2.5 million Daltons. High molecular weight HA is commonly used in HA fillers designed to enhance volume. The concentration of HA in a product is usually indicated as milligrams per milliliter (mg/mL), with most products containing between 15 and 25 mg/mL of HA. The most common concentration is around 20 mg/mL. The concentration of hyaluronic acid affects not only the physical properties of the filler, such as strength, but also the degree of swelling after the procedure [13]. Typically, 5.5 mg of HA balances with 1 mL of water, so higher concentration HA fillers can attract more water post‐injection, resulting in greater swelling at the treatment site [8].

As previously explained in the manufacturing process section, many filler manufacturers have long‐established methods for producing safe HA filler products. Although each company may have proprietary techniques to achieve the unique characteristics of their fillers, the basic principles of safe filler production are generally the same. If a new product differs significantly from the traditional manufacturing processes of existing fillers, it should be scrutinized more carefully. When using new products that claim to employ different or innovative technologies, it is advisable to observe their safety over a sufficient period to confirm their reliability.

### 2.4 Biphasic Versus Monophasic Hyaluronic Acid Fillers

HA fillers available on the market are generally classified as either biphasic or monophasic based on their manufacturing methods. While filler manufacturing technology has significantly advanced, leading to the development of HA fillers that claim to combine the advantages of both types, the fundamental concepts remain unchanged. Biphasic HA fillers emphasize physical cross‐linking, whereas monophasic HA fillers emphasize chemical cross‐linking [8].

Understanding these fundamental differences is crucial when choosing a filler product. Biphasic fillers typically consist of HA particles of various sizes that are physically cross‐linked, resulting in a firmer texture and higher lifting capacity. In contrast, monophasic fillers consist of a homogeneous gel with chemically cross‐linked HA, offering a smoother consistency and better cohesiveness [9].

Despite advancements in filler technology, the basic principles of biphasic and monophasic fillers still hold. It is important to be aware of any additional factors that might affect the properties of the fillers, beyond the basic cross‐linking methods, before using the product. Each manufacturing company may incorporate unique elements into their fillers to enhance specific characteristics. Therefore, a thorough understanding and consideration of these factors are essential for selecting the most suitable filler for clinical use.

### 2.5 Rheological Properties

#### 2.5.1 Particle Form

**FIGURE 1:**
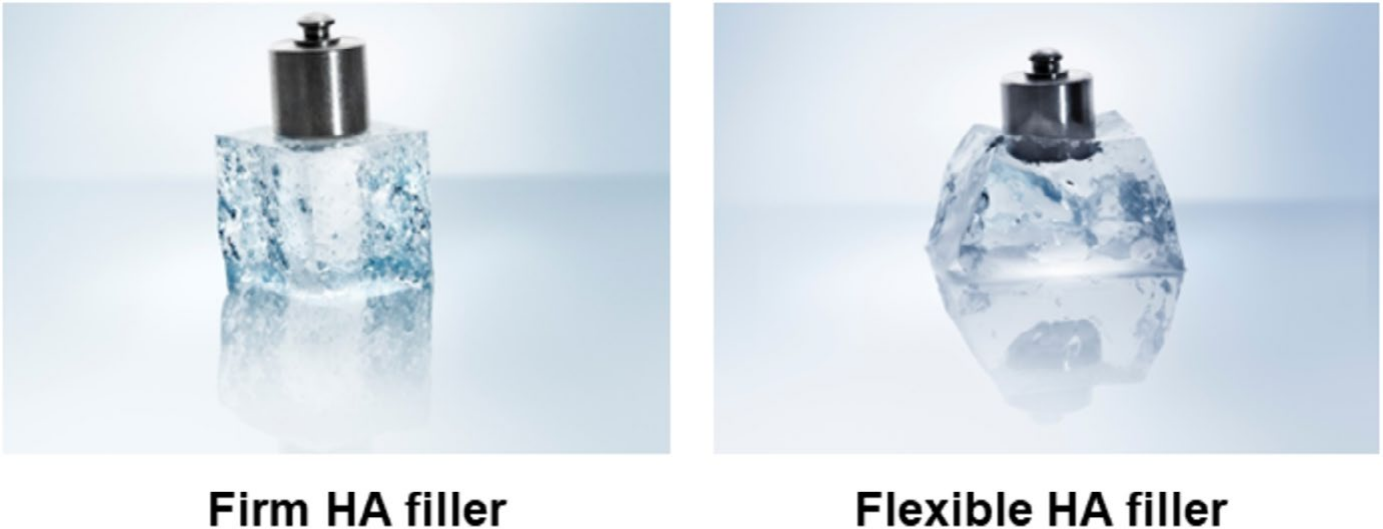
Division of HA filler based on consistency—firm or flexible HA filler.

When discussing the rheological properties of HA fillers, it is common to differentiate between biphasic fillers, which have good lifting capacity, and monophasic fillers, known for their cohesion. A prevalent misconception is that biphasic fillers are composed of particles, while monophasic fillers are uniform gels devoid of particles. However, microscopic examination reveals that all HA fillers consist of gel particles. Monophasic fillers feel smoother to the touch because their particles are less discernible. Therefore, it is more accurate to classify fillers based on their consistency: biphasic fillers possess distinct and firmer particles, whereas monophasic fillers have less noticeable and more flexible particles (Figure 1) [9, 14].

#### 2.5.2 Viscoelasticity

Viscoelasticity, defined by the parameters of elasticity (*G*′ modulus, storage modulus) and viscosity (*G*″ modulus, loss modulus), is a crucial concept in rheology used to assess the properties of materials. These values are combined to calculate the viscoelastic modulus (G*), which provides a comprehensive measure of a material's resistance to external forces and its degree of deformation. HA fillers, as viscoelastic materials, exhibit both elastic and viscous behaviors. The *G*′ value measures the material's resistance to deformation, while the *G*″ value measures how easily the material continues to deform under an external force [15].

Biphasic fillers, with their distinct and firm particles, typically exhibit higher viscoelastic values in laboratory tests, indicating better volume retention and shape maintenance after injection. However, they are also stiffer and more challenging to use. Monophasic fillers, on the other hand, have lower viscoelastic values but are easier to inject due to their smoother consistency.

For monophasic fillers, an increase in viscosity is often accompanied by a proportional increase in elasticity, making it possible to infer viscoelastic properties from the viscoelastic modulus alone. In contrast, biphasic fillers may show high viscoelastic modulus values due to their elasticity, but their absolute viscosity values may not accurately reflect their viscous properties [15].

To gain a more comprehensive understanding of a filler's rheological characteristics, one can compare the values of elasticity and viscosity using the phase angle (tangent δ), which is calculated as the ratio of *G*″ to *G*′ (*G*″/*G*′). A phase angle approaching 0 indicates that the material behaves more like a solid elastic body, whereas a phase angle approaching 1 suggests that the material behaves more like a viscous fluid. For example, even with similar viscosity values, biphasic fillers will exhibit lower phase angles, reflecting their greater elasticity, while monophasic fillers will display higher phase angles due to their relatively higher viscosity [16].

Manufacturers typically measure and provide data on the viscoelastic modulus and phase angle of their HA fillers. It is advisable to request this information to better predict the rheological behavior of the fillers being used.

#### 2.5.3 Cohesiveness and Flexibility

##### 2.5.3.1 Cohesiveness

Cohesiveness refers to the force that enables filler particles to restore their structure after being deformed by external forces. As previously discussed, biphasic and monophasic fillers exhibit distinct cohesiveness characteristics due to their differing manufacturing processes. Generally, it is not advisable to use fillers with low cohesiveness and high elasticity in areas of the face that experience significant movement. These regions are frequently subjected to external forces, necessitating fillers that can gently deform under pressure and then return to their original shape once the pressure is relieved [17, 18].

If a filler possesses high elasticity and resists deformation, patients may experience discomfort when moving their facial muscles. Furthermore, when a filler with high elasticity but low cohesiveness is deformed by strong pressure (e.g., during a massage), it may not revert to its original shape once the pressure is removed, leading to an altered appearance post‐procedure [19].

Therefore, for areas such as the cheeks, monophasic HA fillers with good cohesiveness are ideal. These fillers can maintain a proper shape and respond gently to minor daily forces, such as facial movements or touch, returning to their original form once the force is removed [20].

##### 2.5.3.2 Flexibility

**FIGURE 2:**
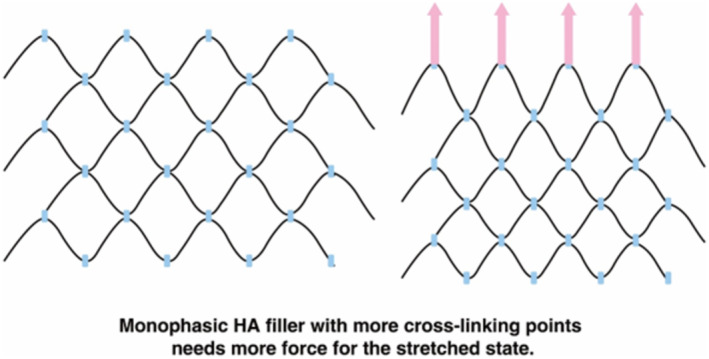
Monophasic HA filler with more cross‐linking points.

Areas around the mouth experience a significant amount of stretching and contracting forces. Hence, it is crucial to choose a monophasic filler with appropriate flexibility that can adapt well to such forces [21, 22]. Chemical cross‐linking does not necessarily result in high cohesiveness, and heavily cross‐linked monophasic HA fillers may not demonstrate good flexibility. Fillers that have undergone extensive chemical cross‐linking to increase stickiness may have HA molecules bound closely together, thereby diminishing their capacity to stretch and contract smoothly under lateral forces (Figure 2).

**FIGURE 3:**
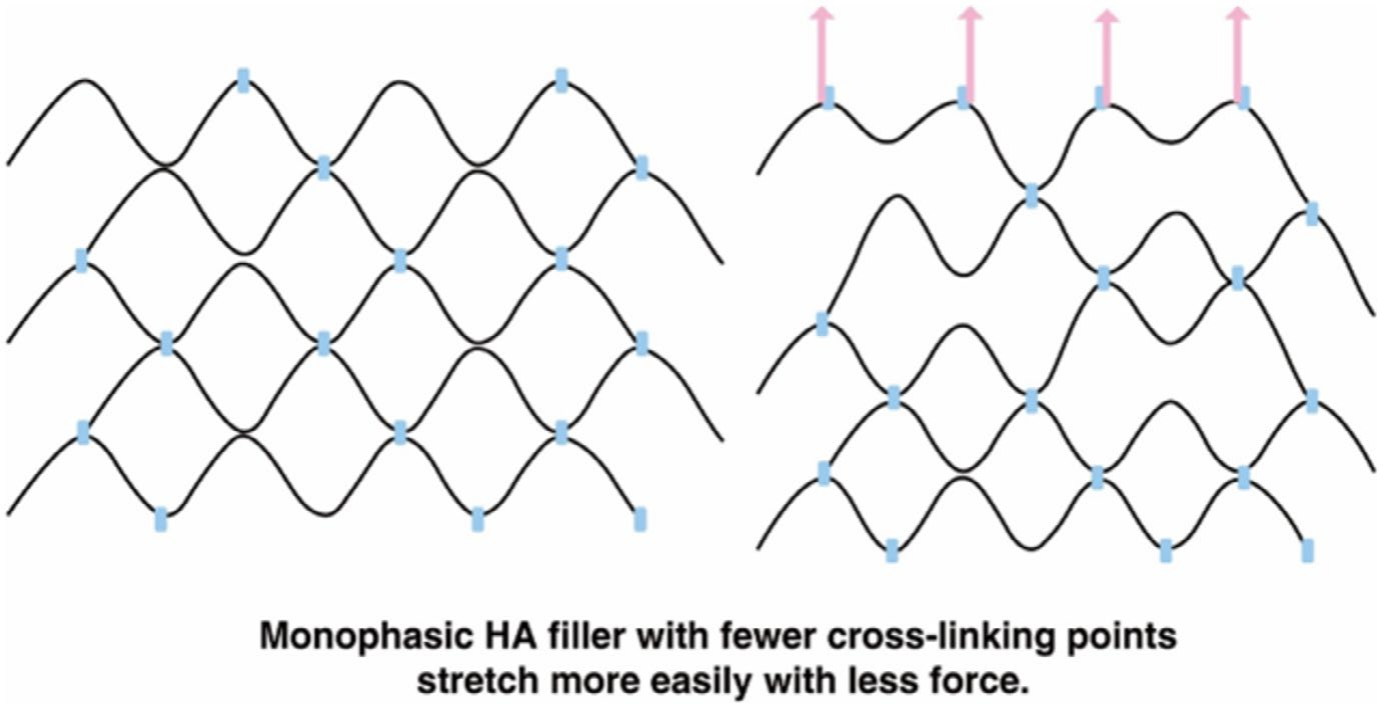
Monophasic HA filler with fewer cross‐linking points.

In contrast, monophasic HA fillers with less densely packed HA molecules display more fluid‐like properties, allowing them to stretch and contract more easily under the same force, demonstrating better adaptability (Figure 3). Therefore, for areas with significant movement around the mouth, it is preferable to use monophasic fillers that offer not just good cohesiveness but also sufficient flexibility to accommodate the skin's stretching and contracting without breaking the filler's basic structure. This ensures better outcomes and less discomfort for patients, particularly in high‐movement areas around the mouth.

In recent times, there has been a trend toward selecting fillers based on the specific treatment area, considering the required viscoelasticity, cohesiveness, and flexibility to choose the most suitable product.

#### 2.5.4 Particle Size and Consistency

##### 2.5.4.1 Particle Size

The particle size of HA fillers varies depending on the manufacturing methods employed by each company. Fillers composed of larger particles are generally more advantageous for creating a more prominent volume. Additionally, the consistency of particle size has become increasingly important, as uniform particle sizes make it easier to achieve a more even overall shape. When injected with consistent pressure, fillers with uniform particle sizes are smoother to administer compared to those with irregular particle sizes. Thus, fillers with consistent particle sizes retain the necessary viscoelastic properties while being more user‐friendly [23].

### 2.6 Extrusion Force and Molding

Improving the consistency of particle size does not reduce the viscoelastic values but decreases the required extrusion force. This enhancement makes the fillers easier to use while maintaining similar volumizing capabilities. Another practical advantage of consistent particle sizes is the ease with which the filler can be molded into the desired shape post‐injection. Fillers with irregular particle sizes are more challenging to mold compared to those with uniform particle sizes [24].

**FIGURE 4:**
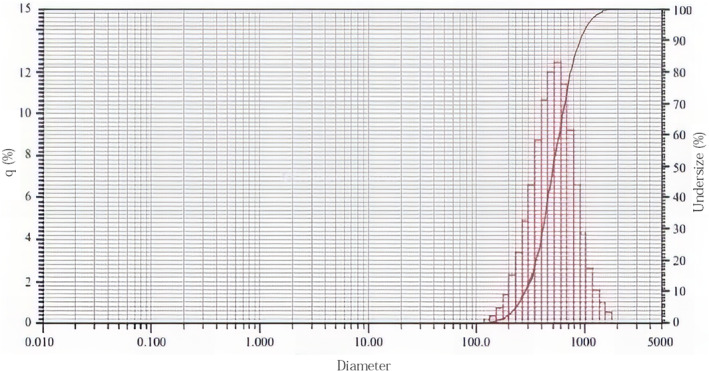
Particle size of the HA filler product.

The following graph (Figure 4) illustrates the particle sizes of domestically produced HA filler products. An optimal filler should have a narrow diameter range with minimal spread. By enhancing the consistency of filler particles through homogenization processes, it is possible to achieve similar volumizing effects while improving ease of use. Fillers with consistent particle sizes and shapes can be more effectively employed in cases or treatment areas that were previously difficult to manage with fillers composed of irregular particles [25].

### 2.7 Recommendation

**FIGURE 5:**
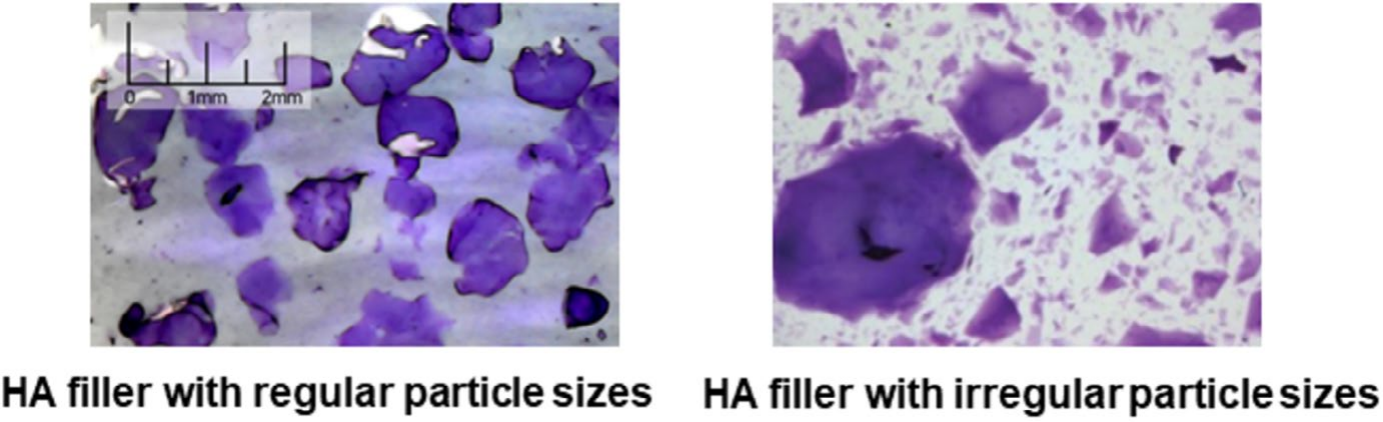
Comparison of particle size uniformity of HA fillers.

When selecting a product, it is advisable to check the particle size and consistency of the filler being used. Ensuring that the filler has uniform particle sizes will not only make the injection process smoother but also facilitate better shaping and molding outcomes post‐injection (Figure 5).

### 2.8 Volume Maintenance After Injection

To facilitate easy injection, molding, and shape creation with minimal foreign body sensation, some HA fillers are mixed with a free HA solution. These fillers are designed to be softer and more manageable during and after the procedure. However, as the free HA is broken down and absorbed a few days post‐injection, the overall volume of the injected filler decreases, leading to a reduction in volume over several weeks.

Several factors influence the maintenance of shape and volume after the filler injection. It is crucial for practitioners to understand how well the filler they are using will retain its volume over time. Awareness of these factors and the properties of the chosen filler can help predict the longevity of the filler's effect and manage patient expectations effectively.

### 2.9 Injection Pressure

The injection pressure required for HA fillers typically tends to be more consistent with monophasic fillers compared to biphasic fillers, which can exhibit more variability [26]. Although manufacturers provide data on the force needed to inject their fillers, practitioners should personally assess the required injection pressure during actual use. When a force is applied to an object, the change in its length in the direction of the force relative to the changes in the perpendicular directions is described by Poisson's ratio. Generally, rigid solids have a Poisson's ratio of less than 0.3, while viscoelastic materials such as rubber have a ratio of about 0.5. A higher ratio indicates that the material deforms more easily under compression or tension [27].

When pressure is applied to expel filler from a syringe, the material adheres to these principles. If the filler has high viscosity and large particle size, it will not easily exit through the syringe's tip and will instead press against the syringe walls, requiring more force and making it harder to use.

**FIGURE 6:**
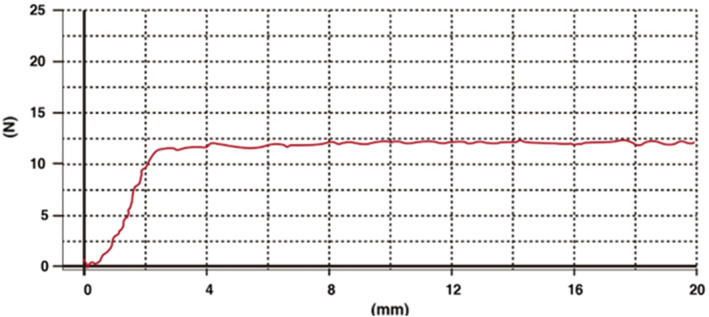
Graphical appearance of the desirable injection force of the HA filler product.

To assess the required injection pressure for a filler, connect the supplied needle and apply minimal force to the plunger to expel the filler. Measure the force needed for the filler to start emerging from the needle and evaluate the pressure needed for continuous injection. A filler that requires consistent pressure without fluctuations is considered easy to inject (Figure 6) [28].

**FIGURE 7:**
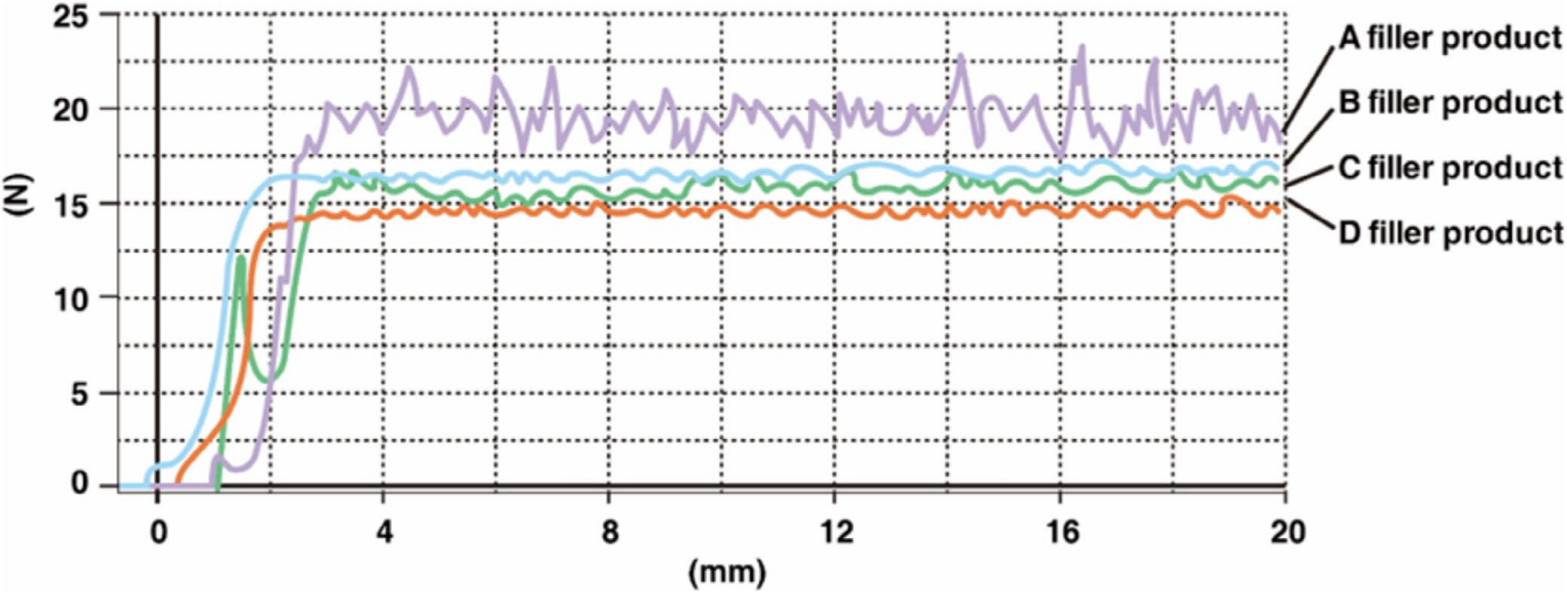
Comparison of injection force of various HA filler products.

The study of the authors demonstrates that Figure 7 illustrates the measured injection pressures required for various HA fillers. Filler A demonstrates the most inconsistent and irregular pressure requirements, making it less favorable. Comparatively, while B and C require slightly higher pressures, B exhibits more consistent pressure requirements overall. Filler D, with the lowest and most consistent pressure requirements, is deemed the easiest HA filler to use.

### 2.10 Presence of Additives

Initially, HA fillers were produced without additives. However, modern HA fillers commonly include 3% lidocaine to reduce pain during the procedure [29, 30, 31]. Manufacturers must ensure that the addition of lidocaine does not alter the physical properties of the filler.

In addition to lidocaine, some HA fillers now include vitamins and antioxidants [32]. Antioxidants are believed to inhibit the activation of reactive oxygen species, which can cause osmotic degradation during the initial filler injection. Therefore, some manufacturers add antioxidants like mannitol to extend the longevity of the HA filler. Mannitol is said to inhibit the degradation of fillers caused by free radicals and possesses antibacterial properties. However, as mannitol is a polymer substance that attracts water, it can cause the filler to increase in volume beyond the initial amount injected [33]. This potential for increased volume should be taken into consideration when using such fillers.

### 2.11 Degree of Dialysis or Washing

Dialysis or washing is a crucial process to remove toxic substances such as sodium hydroxide (NaOH), residual butanediol diglycidyl ether (BDDE), and endotoxins from fillers. The removal of these toxic substances from the HA gel, which is solid in form, takes a considerable amount of time, and each manufacturer employs their own specific methods for dialysis or washing. Generally, a cycle of dialysis or washing is considered to be 24 h long, but there is no standardized protocol for the number of cycles required. We must rely on the manufacturers' descriptions of their processes and their data regarding residual BDDE and endotoxin levels [34].

Even if a product meets the regulatory standards set by health authorities like the Food and Drug Administration (FDA) or the Korean Food and Drug Administration (KFDA), it does not guarantee the complete absence of toxic substances. Paradoxically, this means that adverse reactions related to these substances can still occur with approved filler products.

If a particular filler product has a higher rate of early infections, hypersensitivity, or tissue reactions compared to others, it is important to determine whether the product is at fault. The following steps should be taken to check for product anomalies:

#### 2.11.1 Evaluate Immediate Post‐Procedure Reactions

Monitor for any immediate adverse reactions following the procedure, including inflammation, allergic responses, severe bleeding, or, in extreme cases, necrosis or blindness due to intravascular injection.

#### 2.11.2 Assess Delayed Reactions

Be aware of delayed reactions that can occur more than a month post‐procedure, sometimes even up to a year later. These may include unexpected inflammatory responses and could be due to the patient's immune system, latent infections, or issues with the product itself.

#### 2.11.3 Check Consistency Across Batches

If similar adverse reactions are observed across multiple patients, it may be necessary to check the consistency of the product across different batches. Consulting with nearby clinics using the same product can help determine whether the issue is widespread.

#### 2.11.4 Reevaluate Technique and Protocol

Ensure that the technique and procedural protocol are not contributing to the adverse effects. If the problem persists, discuss with the manufacturer about recalling or exchanging products from the affected batch.

Regular monitoring and thorough evaluation of both immediate and delayed reactions are essential for maintaining patient safety and ensuring the quality of filler products used in clinical practice.

### 2.12 Adverse Effect Monitoring

**FIGURE 8:**
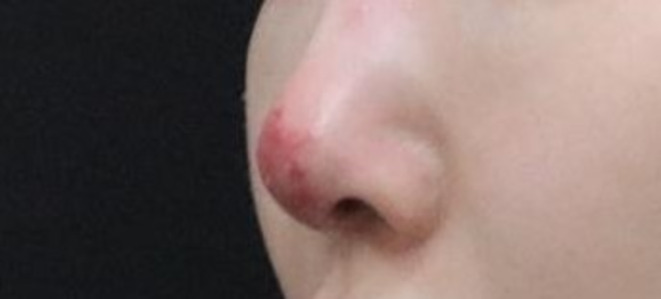
Ischemia of the nasal tip after filler injection.

Adverse effects from filler procedures can be categorized based on their onset time into immediate and delayed reactions [35]. Immediate adverse effects occur right after the procedure and include tissue reactions to residual or toxic substances within the filler, allergic responses, severe bleeding, and in extreme cases, tissue necrosis or blindness due to intravascular injection (Figure 8) [8].

In contrast, delayed adverse effects may manifest more than a month post‐injection, sometimes even up to a year later. These can include unexplained inflammatory reactions. Identifying the exact cause of delayed adverse effects is challenging, as they may stem from issues with the patient's immune system, latent infections, or problems inherent to the product itself.

When delayed adverse effects occur, both the medical practitioner and the patient may find it difficult to determine the cause. In such cases, the practitioner should immediately cease using the suspected product and investigate the cause. It is also important to check if other patients who received the same product are experiencing similar issues. Consulting with nearby clinics using the same product can help determine if the problem is widespread [8, 36].

If adverse effects are determined not to be attributable to the product but rather linked to the practitioner's technique or procedure, it is imperative to reassess and enhance these methods. Conversely, if the issue is traced back to the product itself, the practitioner should discuss the potential for recalling or replacing products from the affected batch with the manufacturer.

Prematurely employing a product in large quantities before confirming its safety can complicate the management of delayed adverse effects. Hence, it is crucial to observe newly released filler products for an adequate period, ideally at least 6 months, to monitor for any adverse effects before widespread use.

Routine and meticulous monitoring of both immediate and delayed reactions is vital to ensure patient safety and uphold the standard of filler products employed in clinical practice.

### 2.13 Advance Uses of Hyaluronic Acid Filler

Hyaluronic acid (HA) fillers with low levels of cross‐linking have become popular as skin boosters, primarily for enhancing hydration and skin quality through intradermal and subdermal injections. Unlike heavily cross‐linked fillers designed for volumizing, low‐cross‐linked HA fillers are lighter and more fluid, making them ideal for improving skin texture and elasticity rather than adding volume. These fillers retain the natural properties of HA, which is already abundant in the skin's dermal layer, helping retain moisture, supporting hydration, and stimulating growth factors. As a result, HA skin boosters provide a more natural, subtle improvement by enhancing the skin's ability to hold water, which in turn smooths and softens the skin's surface [37].

In clinical settings, HA fillers with low cross‐linking are used to address skin dryness, loss of elasticity, and fine lines. They act as “moisturizers” at a deeper level, delivering hydration directly to the dermis and subdermal layers, where each HA molecule can bind up to 218 water molecules. This effect is particularly beneficial in thin or delicate skin areas, such as around the eyes, where added volume is not the primary goal. Studies have shown that injecting these fillers encourages collagen synthesis, as HA pulls in water from the extracellular matrix, increasing skin volume by up to 500–1000 times its molecular size. This expansion leads to fibroblast activation, which further promotes collagen production, thereby supporting a healthier, plumper skin structure [38].

Low‐cross‐linked HA skin boosters are widely used due to their smooth integration into peripheral tissues, resulting in a natural finish without surface irregularities. They have a shorter duration than more cross‐linked fillers, typically lasting around 6 months, and are often administered in a series of three sessions for optimal results. Recent advances, such as combining HA with glycerol (as seen in Belotero Revive), have extended the benefits, enhancing skin hydration, firmness, and glow for up to 36 weeks. These innovations continue to push the boundaries of HA skin boosters, creating formulations that not only improve skin appearance but also support the skin's resilience and barrier function [38].

Recent advancements in self‐crosslinking hyaluronic acid (SC‐HA) fillers represent a significant innovation in minimally invasive aesthetic treatments, aiming to provide longer‐lasting and more effective outcomes. Unlike traditional fillers that require chemical cross‐linking agents, SC‐HA fillers undergo a natural cross‐linking process facilitated by active oxygen within the body. This process, achieved through gallol modification of the hyaluronic acid structure, allows for immediate gelation upon injection, forming a stable and volumizing matrix. This eliminates the need for potentially toxic cross‐linking agents, enhancing both the safety and biocompatibility of the filler [39].

### 2.14 Non‐HA Filler

Non‐hyaluronic acid (non‐HA) fillers, including poly‐L‐lactic acid (PLLA), calcium hydroxylapatite (CaHA), and polymethyl methacrylate (PMMA), provide unique alternatives to HA fillers, each possessing distinctive properties that address specific aesthetic needs beyond the scope of HA fillers alone. Poly‐L‐lactic acid (PLLA) functions as a biostimulatory filler that gradually stimulates collagen production rather than delivering immediate volumization. Its effects emerge progressively over several months as collagen forms around the injected particles, yielding a natural, long‐lasting volumizing outcome. PLLA is particularly effective for patients seeking gradual enhancement in volume‐deficient areas, such as the cheeks and temples, though its application requires skilled technique and careful patient selection to mitigate risks like nodule formation and uneven contouring [40, 41].

Poly‐D, L‐Lactic Acid (PDLLA) is a biocompatible, biodegradable polymer used in fillers like Juvelook for its robust collagen‐stimulating effects and known to provide less risk of nodule formation. PDLLA provides both immediate and sustained rejuvenation by inducing collagen production, which gradually restores volume and firmness in targeted areas. This collagen response improves skin elasticity, smooths fine lines, and enhances overall skin texture, with results lasting up to 18–24 months. PDLLA is particularly suitable for areas needing structural support, such as the cheeks, nasolabial folds, and jawline, offering a subtle, natural‐looking enhancement. Due to its biocompatible degradation into harmless byproducts, PDLLA is a safe and effective option, appealing to patients seeking non‐surgical anti‐aging treatments [42, 43].

Calcium hydroxylapatite (CaHA), composed of calcium‐based microspheres in a gel carrier, provides immediate volume upon injection along with collagen‐stimulating effects over time. With a denser consistency, CaHA is ideal for deeper injections in areas requiring structural support, such as the jawline and cheeks. As a biodegradable filler, CaHA generally maintains its effects for over a year but is less suitable for delicate areas like the lips or under‐eye regions, where its density could lead to undesired outcomes [44]. Polymethyl methacrylate (PMMA) is a semi‐permanent filler consisting of smooth microspheres suspended in collagen, providing durable volume correction [45, 46]. The PMMA microspheres act as a scaffold, promoting collagen formation around them, and are commonly used in areas like the nasolabial folds. However, PMMA's permanence necessitates precise application and is generally recommended for experienced practitioners and patients seeking long‐term results. Each type of non‐HA filler presents distinct advantages and limitations, and optimal filler selection requires consideration of the patient's aesthetic goals, target area, and desired duration of effects. While HA fillers are versatile and reversible, non‐HA fillers offer extended duration and collagen‐stimulating benefits, making them valuable options for patients with specific aesthetic needs.

## 3 Discussion

High‐viscosity fillers are generally unsuitable for superficial fat tissues, as their dense consistency can limit even distribution and exert undue pressure on surrounding structures, such as the lip elevators. Instead, these fillers should be strategically placed within the deep fat tissues, where they can provide stable volume support without affecting muscle movement or causing uneven texture on the skin's surface. This deeper placement allows for a more natural contour and avoids complications related to filler spread and pressure on delicate areas.

Misuse of hyaluronic acid fillers, particularly when high‐viscosity fillers are placed superficially or in excessive amounts, can lead to a condition known as “overfilled syndrome.” This occurs when too much filler is injected or placed incorrectly, resulting in an unnatural, overly plump appearance, distortion of facial features, and sometimes impaired movement. Overfilled syndrome can cause aesthetic concerns, such as a heavy or bloated look, and may impact facial expressions due to excess pressure on surrounding muscles and tissues. Proper technique and filler selection, based on tissue depth and patient anatomy, are essential to prevent this outcome and achieve natural‐looking results.

**FIGURE 9:**
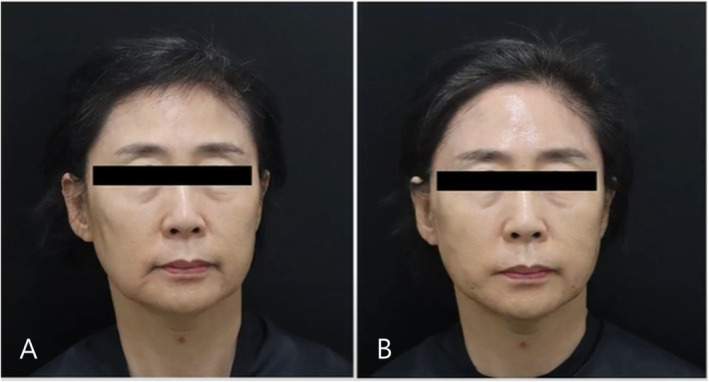
The patient had a flat forehead with a prominent supraorbital ridge; therefore, 3 cc of HA filler with high viscosity and high elasticity was injected above the ridge. The image shows the forehead before (A) and after (B) the treatment.

**FIGURE 10:**
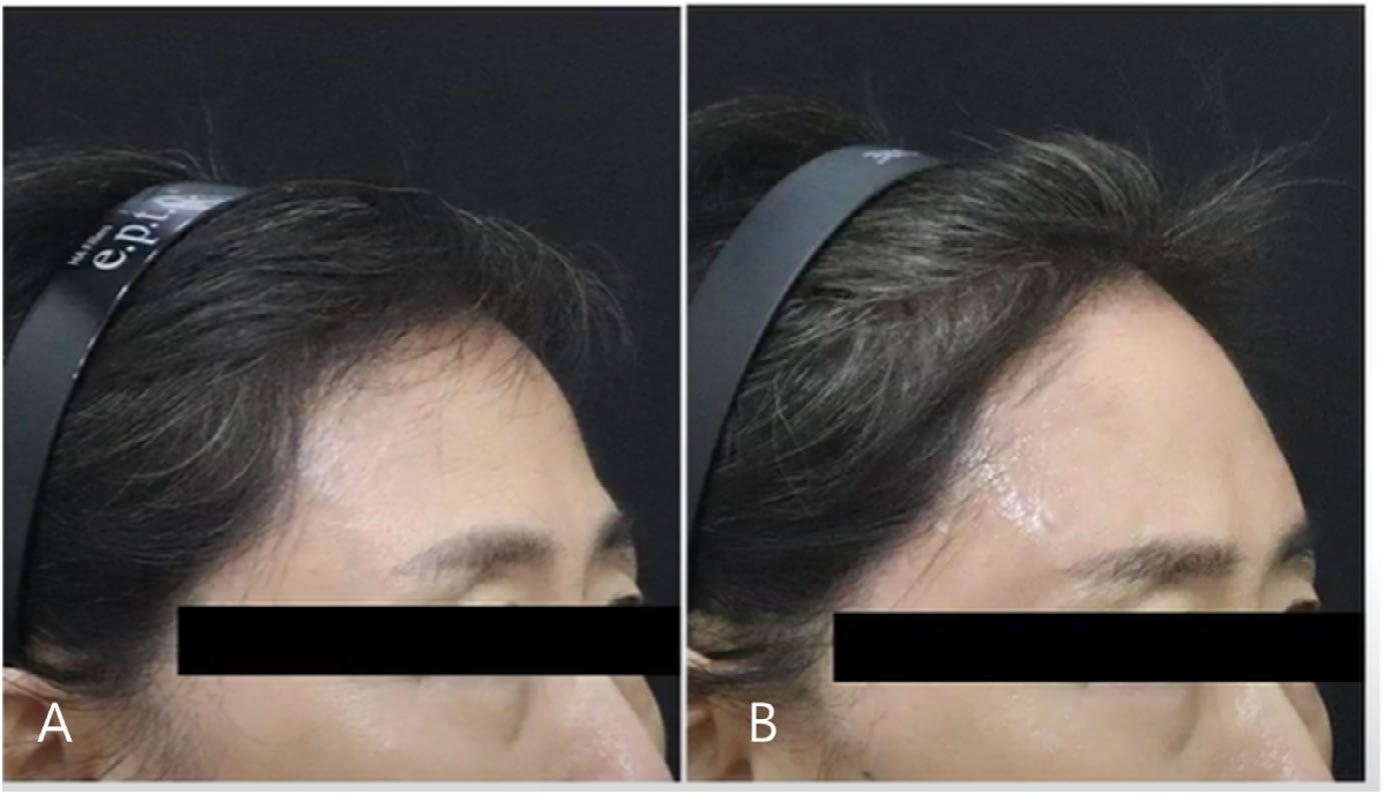
The patient received 3 cc of high‐viscosity and high‐elasticity HA filler injected above the ridge. The image is taken from a 45° angle, showing before (A) and after (B) the treatment.

Selecting appropriate fillers in aesthetic medicine involves considering rheological properties, safety profiles, and clinical efficacy. Understanding these factors is crucial for optimizing patient outcomes and minimizing adverse effects (Figures 9 and 10). HA fillers are recommended for novice practitioners due to their reversibility and established safety profiles, which allow for dissolution with hyaluronidase in case of adverse outcomes. As clinicians gain experience, they may explore non‐HA fillers for longer‐lasting results, but caution is advised due to their irreversibility and potential for long‐term complications [6, 7].

**FIGURE 11:**
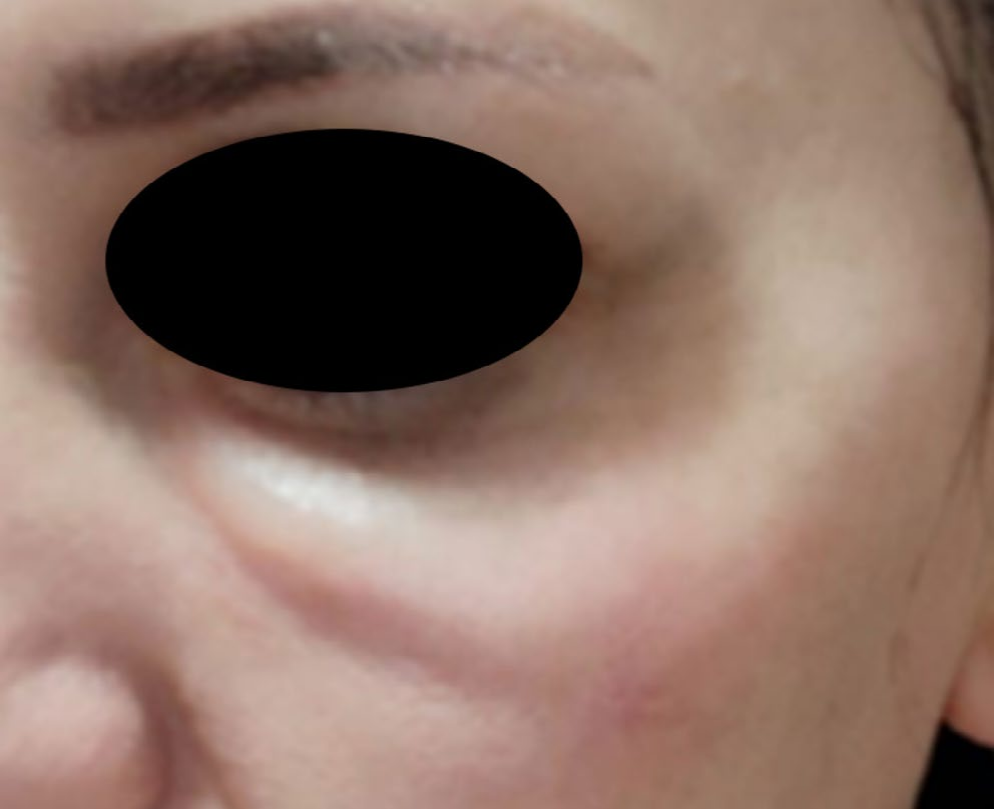
Incorrect method: High *G*′ filler injected superficially, resulting in the Tyndall effect and a mass‐like appearance.

**FIGURE 12:**
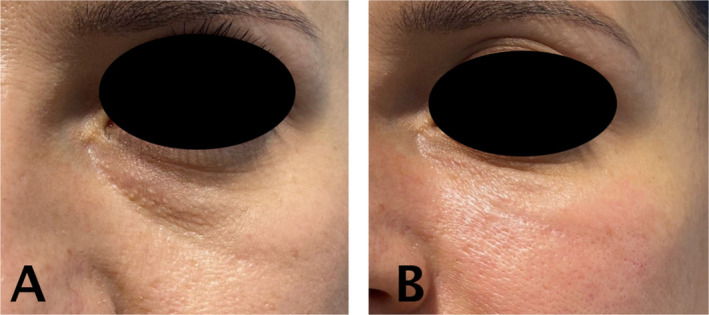
Correct method: Filler injected deeply. Before treatment (A) and after treatment (B).

For example, when addressing hollow areas under the eyes, softer fillers are typically used. However, such fillers tend to inflate the skin during the procedure, often leading to swelling and a higher likelihood of the Tyndall effect. Instead, placing a firmer filler precisely beneath the muscle along the groove, while preserving the tough ligamentous structures in the under‐eye area, can result in smoother skin and minimize the risk of the Tyndall effect. This demonstrates that contrary to conventional assumptions, selecting the physical properties of fillers according to the specific characteristics of each facial region is crucial (Figures 11 and 12).

Understanding the differences between biphasic and monophasic HA fillers is also essential. Biphasic fillers, with their particle‐based structure, provide strong lifting capacity but can be challenging to inject and mold. In contrast, monophasic fillers offer smoother consistency and better cohesiveness, making them easier to handle and suitable for dynamic facial areas [26, 47]. The viscoelastic properties of fillers, including parameters like *G*′ and *G*″ moduli and phase angle, play a critical role in their performance [48, 49]. Clinicians should analyze these rheological data from manufacturers to make informed decisions tailored to patient needs and treatment areas. This approach enhances the likelihood of achieving satisfactory aesthetic results while minimizing complications.

## Author Contributions

Conceptualization: Gi‐Woong Hong, Kyu‐Ho Yi, Jovian Wan; writing – original draft preparation: Gi‐Woong Hong, Jovian Wan, Song‐Eun Yoon, Sky Wong; writing – review and editing: Gi‐Woong Hong, Kyu‐Ho Yi, Song‐Eun Yoon, Sky Wong; visualization: Gi‐Woong Hong and Kyu‐Ho Yi; supervision: Gi‐Woong Hong and Kyu‐Ho Yi. All authors have reviewed and approved the article for submission.

## Disclosure

The authors have nothing to report.

## Conflicts of Interest

The authors declare no conflicts of interest.

## Data Availability

The data that support the findings of this study are available from the corresponding author upon reasonable request.

## References

[1] G. Murray , C. Convery , L. Walker , and E. Davies , “Guideline for the Safe Use of Hyaluronidase in Aesthetic Medicine, Including Modified High‐Dose Protocol,” Journal of Clinical and Aesthetic Dermatology 14, no. 8 (2021): E69–E75. PMC8570661 34840662

[2] H. Jung , “Hyaluronidase: An Overview of Its Properties, Applications, and Side Effects,” Archives of Plastic Surgery 47, no. 4 (2020): 297–300. 32718106 10.5999/aps.2020.00752 PMC7398804

[3] O. R. Olaiya , D. Forbes , S. Humphrey , K. Beleznay , M. Mosher , and J. Carruthers , “Hyaluronidase for Treating Complications Related to HA Fillers: A National Plastic Surgeon Survey,” Plastic Surgery 30, no. 3 (2022): 233–237. 35990397 10.1177/22925503211019618 PMC9389064

[4] G. Kroumpouzos and P. Treacy , “Hyaluronidase for Dermal Filler Complications: Review of Applications and Dosage Recommendations,” JMIR Dermatology 7 (2024): e50403. 38231537 10.2196/50403 PMC10836581

[5] G. Zaccaria , A. Dotti , E. Benanti , C. Vigliarolo , and L. Vaienti , “A Treatment Algorithm for Hyaluronic Acid Filler Related Complications of the Face,” Journal of Plastic, Reconstructive & Aesthetic Surgery 91 (2024): 207–217. 10.1016/j.bjps.2024.02.010 38422922

[6] L. N. Trinh and A. Gupta , “Non‐Hyaluronic Acid Fillers for Midface Augmentation: A Systematic Review,” Facial Plastic Surgery 37, no. 4 (2021): 536–542. 33648015 10.1055/s-0041-1725164

[7] D. Thioly‐Bensoussan , “Non‐Hyaluronic Acid Fillers,” Clinics in Dermatology 26, no. 2 (2008): 160–176. 18472057 10.1016/j.clindermatol.2007.09.017

[8] D. Funt and T. Pavicic , “Dermal Fillers in Aesthetics: An Overview of Adverse Events and Treatment Approaches,” Clinical, Cosmetic and Investigational Dermatology 6 (2013): 295–316. 24363560 10.2147/CCID.S50546 PMC3865975

[9] S. P. Fundarò , G. Salti , D. M. H. Malgapo , and S. Innocenti , “The Rheology and Physicochemical Characteristics of Hyaluronic Acid Fillers: Their Clinical Implications,” International Journal of Molecular Sciences 23, no. 18 (2022): 10518, 10.3390/ijms231810518. 36142430 PMC9503994

[10] M. Dovedytis , Z. J. Liu , and S. Bartlett , “Hyaluronic Acid and Its Biomedical Applications: A Review,” Engineered Regeneration 1 (2020): 102–113.

[11] K. Al‐Ghanim , R. Richards , and S. Cohen , “A Practical Guide to Selecting Facial Fillers,” Journal of Cosmetic Dermatology 22, no. 12 (2023): 3232–3236. 37395390 10.1111/jocd.15867

[12] S. H. Dayan and B. A. Bassichis , “Facial Dermal Fillers: Selection of Appropriate Products and Techniques,” Aesthetic Surgery Journal 28, no. 3 (2008): 335–347. 19083546 10.1016/j.asj.2008.03.004

[13] G. CQ , J. J. EC , G. CL , X. A. PM , and G. A , “Hyaluronic Acid‐Extraction Methods, Sources and Applications,” Polymers 15, no. 16 (2023): 3473. 37631529 10.3390/polym15163473 PMC10459667

[14] K. Y. Park , H. K. Kim , and B. J. Kim , “Comparative Study of Hyaluronic Acid Fillers by In Vitro and In Vivo Testing,” Journal of the European Academy of Dermatology and Venereology 28, no. 5 (2014): 565–568. 23495913 10.1111/jdv.12135

[15] L. Budai , M. Budai , Z. E. Fülöpné Pápay , Z. Vilimi , and I. Antal , “Rheological Considerations of Pharmaceutical Formulations: Focus on Viscoelasticity,” Gels 9, no. 6 (2023): 469, 10.3390/gels9060469. 37367140 PMC10298452

[16] N. Zerbinati , M. C. Capillo , S. Sommatis , et al., “Rheological Investigation as Tool to Assess Physicochemical Stability of a Hyaluronic Acid Dermal Filler Cross‐Linked With Polyethylene Glycol Diglycidyl Ether and Containing Calcium Hydroxyapatite, Glycine and L‐Proline,” Gels 8, no. 5 (2022): 264. 35621562 10.3390/gels8050264 PMC9140203

[17] K. T. Kim , W. Lee , and E. J. Yang , “Cohesiveness of Hyaluronic Acid Fillers: Evaluation Using Multiple Cohesion Tests,” Archives of Plastic Surgery 51, no. 1 (2024): 14–19. 38425852 10.1055/a-2234-1019 PMC10901597

[18] A. D. Prasetyo , W. Prager , M. G. Rubin , E. A. Moretti , and A. Nikolis , “Hyaluronic Acid Fillers With Cohesive Polydensified Matrix for Soft‐Tissue Augmentation and Rejuvenation: A Literature Review,” Clinical, Cosmetic and Investigational Dermatology 9 (2016): 257–280. 27660479 10.2147/CCID.S106551 PMC5021061

[19] G. Salti and R. Rauso , “Facial Rejuvenation With Fillers: The Dual Plane Technique,” Journal of Cutaneous and Aesthetic Surgery 8, no. 3 (2015): 127–133. 26644734 10.4103/0974-2077.167264 PMC4645140

[20] J. van Loghem , S. Sattler , G. Casabona , et al., “Consensus on the Use of Hyaluronic Acid Fillers From the Cohesive Polydensified Matrix Range: Best Practice in Specific Facial Indications,” Clinical, Cosmetic and Investigational Dermatology 14 (2021): 1175–1199. 34526796 10.2147/CCID.S311017 PMC8435881

[21] S. Pierre , S. Liew , and A. Bernardin , “Basics of Dermal Filler Rheology,” Dermatologic Surgery 41, no. Suppl 1 (2015): S120–S126. 25828036 10.1097/DSS.0000000000000334

[22] T. Michaud , “Rheology of Hyaluronic Acid and Dynamic Facial Rejuvenation: Topographical Specificities,” Journal of Cosmetic Dermatology 17, no. 5 (2018): 736–743. 30311427 10.1111/jocd.12774

[23] J. Guo , W. Fang , and F. Wang , “Injectable Fillers: Current Status, Physicochemical Properties, Function Mechanism, and Perspectives,” RSC Advances 13, no. 34 (2023): 23841–23858. 37577103 10.1039/d3ra04321e PMC10413051

[24] W. Zhou , S. Hou , S. Deng , et al., “The Intrinsic Relation Between the Hydrogel Structure and In Vivo Performance of Hyaluronic Acid Dermal Fillers: A Comparative Study of Four Typical Dermal Fillers,” Tissue Engineering and Regenerative Medicine 20, no. 3 (2023): 503–517. 37041433 10.1007/s13770-023-00533-0 PMC10219904

[25] A. Nagrale , S. Nevrekar , S. Kawle , H. Gawande , J. Gupte , and S. Gaikwad , “Influence of Filler Particle Sizes on the Physical Properties of Bulk‐Fill Composites Compared to Conventional Composites,” Cureus 15, no. 3 (2023): e36032. 37056541 10.7759/cureus.36032 PMC10089640

[26] Y. Huang , Y. Zhang , X. Fei , Q. Fan , and J. Mao , “Monophasic and Biphasic Hyaluronic Acid Fillers for Esthetic Correction of Nasolabial Folds: A Meta‐Analysis of Randomized Controlled Trials,” Aesthetic Plastic Surgery 46, no. 3 (2022): 1407–1422. 35066619 10.1007/s00266-021-02729-y

[27] K. Masouras , N. Silikas , and D. C. Watts , “Correlation of Filler Content and Elastic Properties of Resin‐Composites,” Dental Materials 24, no. 7 (2008): 932–939. 18155132 10.1016/j.dental.2007.11.007

[28] Y. Lee , S. M. Oh , W. Lee , and E. J. Yang , “Comparison of Hyaluronic Acid Filler Ejection Pressure With Injection Force for Safe Filler Injection,” Journal of Cosmetic Dermatology 20, no. 5 (2021): 1551–1556. 33713373 10.1111/jocd.14064

[29] H. Raspaldo , K. De Boulle , and P. M. Levy , “Longevity of Effects of Hyaluronic Acid Plus Lidocaine Facial Filler,” Journal of Cosmetic Dermatology 9, no. 1 (2010): 11–15. 20367667 10.1111/j.1473-2165.2010.00481.x

[30] S. H. Huang and T. F. Tsai , “Safety and Effectiveness of Hyaluronic Acid Fillers With Lidocaine for Full‐Face Treatment in Asian Patients,” Journal of Drugs in Dermatology 19, no. 9 (2020): 836–842. 33026748 10.36849/JDD.2020.10.36849/JDD.2020.5374

[31] C. Wang , S. Luan , A. C. Panayi , M. Xin , B. Mi , and J. Luan , “Effectiveness and Safety of Hyaluronic Acid Gel With Lidocaine for the Treatment of Nasolabial Folds: A Systematic Review and Meta‐Analysis,” Aesthetic Plastic Surgery 42, no. 4 (2018): 1104–1110. 29740661 10.1007/s00266-018-1149-3

[32] K. Y. Park , J. Seok , E. J. Ko , B. J. Kim , M. N. Kim , and C. S. Youn , “Hyaluronic Acid Filler Combined With Antioxidants for Infraorbital Rejuvenation: Report of Two Cases,” Dermatologic Therapy 30, no. 2 (2017): e12448. 10.1111/dth.12448 28133882

[33] P. André and F. Villain , “Free Radical Scavenging Properties of Mannitol and Its Role as a Constituent of Hyaluronic Acid Fillers: A Literature Review,” International Journal of Cosmetic Science 39, no. 4 (2017): 355–360. 28027572 10.1111/ics.12386

[34] C. H. Jeong , D. H. Kim , J. H. Yune , et al., “In Vitro Toxicity Assessment of Crosslinking Agents Used in Hyaluronic Acid Dermal Filler,” Toxicology In Vitro 70 (2021): 105034. 33096205 10.1016/j.tiv.2020.105034

[35] E. Haneke , “Adverse Effects of Fillers,” Dermatologic Therapy 32, no. 2 (2019): e12676. 30187592 10.1111/dth.12676

[36] T. Bhojani‐Lynch , “Late‐Onset Inflammatory Response to Hyaluronic Acid Dermal Fillers,” Plastic and Reconstructive Surgery. Global Open 5, no. 12 (2017): e1532. 29632758 10.1097/GOX.0000000000001532 PMC5889432

[37] L. Kleine‐Börger , M. Hofmann , and M. Kerscher , “Microinjections With Hyaluronic Acid in Combination With Glycerol: How Do They Influence Biophysical Viscoelastic Skin Properties?,” Skin Research and Technology 28, no. 4 (2022): 633–642. 35643988 10.1111/srt.13167 PMC9907673

[38] D. Hertz‐Kleptow , A. Hanschmann , M. Hofmann , T. Reuther , and M. Kerscher , “Facial Skin Revitalization With CPM‐HA20G: An Effective and Safe Early Intervention Treatment,” Clinical, Cosmetic and Investigational Dermatology 12 (2019): 563–572. 31496779 10.2147/CCID.S209256 PMC6698156

[39] K. H. Yi , S. B. Kim , H. Hu , et al., “Self‐Crossing Hyaluronic Acid Filler With Combination Use of Polydioxanone Thread in Minipig Model,” Journal of Cosmetic Dermatology 23, no. 9 (2024): 2821–2828. 38654663 10.1111/jocd.16338

[40] N. S. Sadick and L. Palmisano , “Case Study Involving Use of Injectable Poly‐L‐Lactic Acid (PLLA) for Acne Scars,” Journal of Dermatological Treatment 20, no. 5 (2009): 302–307. 19340629 10.1080/09546630902817879

[41] H. Lu , H. H. Oh , N. Kawazoe , K. Yamagishi , and G. Chen , “PLLA‐Collagen and PLLA‐Gelatin Hybrid Scaffolds With Funnel‐Like Porous Structure for Skin Tissue Engineering,” Science and Technology of Advanced Materials 13, no. 6 (2012): 064210. 27877537 10.1088/1468-6996/13/6/064210 PMC5099770

[42] S. B. Seo , H. Park , J. Y. Jo , and H. J. Ryu , “Skin Rejuvenation Effect of the Combined PDLLA and Non Cross‐Linked Hyaluronic Acid: A Preliminary Study,” Journal of Cosmetic Dermatology 23, no. 3 (2024): 794–802, 10.1111/jocd.16085. 37969055

[43] K. M. Perez Willis and R. Ramirez Galvez , “Granuloma After the Injection of Poly‐D, L‐Lactic Acid (PDLLA) Treated With Triamcinolone,” Case Reports in Dermatological Medicine 2024 (2024): 6544506. 38698953 10.1155/2024/6544506 PMC11065485

[44] K. Beer , M. Yohn , and J. L. Cohen , “Evaluation of Injectable CaHA for the Treatment of Mid‐Face Volume Loss,” Journal of Drugs in Dermatology 7, no. 4 (2008): 359–366. 18459517

[45] C. C. Medeiros , K. Cherubini , F. G. Salum , and M. A. de Figueiredo , “Complications After Polymethylmethacrylate (PMMA) Injections in the Face: A Literature Review,” Gerodontology 31, no. 4 (2014): 245–250. 23464838 10.1111/ger.12044

[46] R. M. Limongi , J. Tao , A. Borba , et al., “Complications and Management of Polymethylmethacrylate (PMMA) Injections to the Midface,” Aesthetic Surgery Journal 36, no. 2 (2015): 132–135. 26446059 10.1093/asj/sjv195

[47] A. A. Ghaddaf , Y. E. Aljefri , F. A. Alharbi , R. K. Sharif , W. A. Alnahdi , and R. Baaqeel , “Monophasic Versus Biphasic Hyaluronic Acid Filler for Correcting Nasolabial Folds: A Systematic Review and Meta‐Analysis,” Journal of Cosmetic Dermatology 21, no. 2 (2022): 627–635. 34817919 10.1111/jocd.14632

[48] S. Fagien , V. Bertucci , E. von Grote , and J. H. Mashburn , “Rheologic and Physicochemical Properties Used to Differentiate Injectable Hyaluronic Acid Filler Products,” Plastic and Reconstructive Surgery 143, no. 4 (2019): 707e–720e. 10.1097/PRS.0000000000005429 PMC7597953 30921116

[49] G. G. G. Perera , D. F. Argenta , and T. Caon , “The Rheology of Injectable Hyaluronic Acid Hydrogels Used as Facial Fillers: A Review,” International Journal of Biological Macromolecules 268, no. Pt 2 (2024): 131880. 38677707 10.1016/j.ijbiomac.2024.131880

